# Phenotype-first patient matching with SimPheny identifies diagnostic candidates beyond curated gene associations

**DOI:** 10.64898/2026.01.15.26344236

**Published:** 2026-01-17

**Authors:** Isabelle B. Cooperstein, Alistair Ward, Shilpa N. Kobren, Emerson Lebleu, Barry Moore, Rebecca C. Spillmann, Vandana Shashi, Undiagnosed Diseases Network, Gabor T. Marth

**Affiliations:** 1 Department of Human Genetics, University of Utah, Salt Lake City, UT, USA; 2 Frameshift Labs Inc, Cambridge, MA; 3 Department of Biomedical Informatics, Harvard Medical School, Boston, MA; 4 Department of Pediatrics, Division of Medical Genetics, Duke University School of Medicine, Durham, NC

## Abstract

Diagnostic tools for rare diseases typically rely on curated gene-phenotype associations and static disease models, limiting their effectiveness in cases with atypical presentations or previously uncharacterized disorders. To address these limitations, we present SimPheny, a phenotype-first algorithm for gene prioritization that operates independently of documented gene-phenotype associations. SimPheny identifies phenotypically similar diagnosed patients by comparing an undiagnosed patient’s disease presentation to a reference cohort of diagnosed cases, and returns gene hypotheses by matching the undiagnosed patient’s candidate gene list to the causative genes of similar patients using a statistical scoring model. Evaluated in diagnosed probands from the Undiagnosed Diseases Network (UDN) with the true diagnostic gene blinded, SimPheny consistently ranked the diagnostic gene among the top five candidates, outperforming existing tools, particularly for genes with limited gene-phenotype association data. When applied to previously unsolved UDN cases, clinical review confirmed that SimPheny’s high-confidence causative gene predictions were diagnostic in nearly half of the analyzed cases. As the size of the diagnosed reference cohort increases, SimPheny’s diagnostic reach expands without sacrificing ranking performance. By leveraging real patient data rather than curated models, SimPheny provides a generalizable, scalable framework for improving diagnostic yield in rare disease cohorts.

## Introduction

Rare monogenic diseases collectively affect more than 400 million people worldwide, representing a substantial public health challenge [1]. Effective and timely diagnosis of these conditions is therefore essential, both for guiding clinical management and advancing our understanding of disease mechanisms. However, identifying a molecular diagnosis remains challenging for several reasons. More than 80% of the approximately 10,000 recognized rare diseases are presumed to have a genetic basis, yet causal genes or variants have been identified for fewer than half [2, 3]. Even when disease-associated genes are known, the rarity and phenotypic heterogeneity of these disorders often obscure gene-phenotype relationships, complicating recognition of affected individuals [4]. Most patients remain undiagnosed after standard sequencing tests, and many endure years-long diagnostic odysseys marked by uncertainty and repeated misdiagnoses [5, 6]. Together, these challenges underscore the need for improved diagnostic approaches that can resolve rare and heterogeneous disease presentations.

Undiagnosed patients often harbor dozens to hundreds of rare variants that may appear deleterious to gene function; yet, only one or two are typically causative of the disease phenotype [8]. Distinguishing the causal variant(s) remains challenging, particularly for rare or newly implicated genes, where gene-phenotype-disease relationships are incomplete or unknown. In the absence of clear associations linking genes to clinical presentations, evidence falls to variant deleteriousness alone, which provides limited insight into whether disruption of a given gene explains the patient’s phenotype, thereby constraining the ability to resolve gene-level causality [7].

Inconsistent application of phenotypic evidence further hinders variant interpretation, leading to divergent classifications and reduced diagnostic accuracy [9–12]. To address this limitation and harmonize phenotypic representation across rare disease cohorts, international efforts have focused on standardizing phenotype annotation. The Human Phenotype Ontology (HPO) was developed to organize human disease phenotypes into a structured hierarchy, enabling computational analyses of clinical features across patients and diseases [13, 14]. The HPO has been widely adopted in research and clinical settings, supporting gene-phenotype associations in resources such as Orphanet [15, 16] and OMIM [17].

Standardized phenotype terminologies and variant annotations have enabled the development of diagnostic tools that integrate both genotypic and phenotypic evidence. Phenotype-aware variant prioritization tools, such as Exomiser and AI-MARRVEL, combine patient HPO terms with variant pathogenicity scores and allele frequencies to prioritize candidate genes [18–21]. These approaches perform well when causal genes are well-annotated; however, their performance declines when gene-phenotype associations are absent. In such cases, prioritization is based solely on variant-level evidence, which consistently ranks diagnostic variants lower than approaches that integrate genotype and phenotype information [22].

Existing phenotype-first approaches calculate phenotypic similarity between a patient’s HPO terms and curated disease models derived from OMIM or Orphanet to prioritize candidate diseases [21, 23, 24]. While these disease models are invaluable references, they represent idealized summaries that rarely capture the full phenotypic variability observed in real patients. Clinical presentations can vary widely, distorting similarity scores between patients and these “gold-standard” models [25], leading to missed diagnoses in atypical cases. Moreover, patients may present with conditions that are newly emerging, incompletely characterized, or entirely novel, for which curated disease models do not yet exist, fundamentally limiting the diagnostic utility of model-based phenotype matching approaches.

These challenges underscore the need for diagnostic methods that transcend static disease models and instead utilize real-world patient data to more accurately capture the phenotypic diversity and diagnostic complexity of rare diseases. In practice, identifying even a single previously diagnosed patient with a similar phenotypic presentation and overlapping genetic variant(s) can provide decisive evidence for establishing a diagnosis, particularly for atypical or incompletely characterized conditions [26, 27]. Such patient matches provide direct, case-level evidence that can resolve individual diagnoses and refine the known phenotypic spectrum of rare diseases. However, the extreme rarity and geographic dispersion of affected individuals present significant obstacles to implementing patient-to-patient matching at scale.

We present SimPheny, a phenotype-first algorithm that enables scalable patient-to-patient matching based on HPO-encoded phenotypic similarity, followed by integration of genotypic overlap to prioritize candidate genes. Unlike traditional variant-first tools [18–20], SimPheny does not depend on curated disease models or documented gene-phenotype associations. By leveraging real patient data, SimPheny identifies diagnostic candidates that elude existing association-based frameworks, expanding discovery in cases with atypical or incompletely characterized presentations.

We benchmarked SimPheny on a cohort of previously diagnosed probands from the Undiagnosed Diseases Network (UDN) [28], then applied it to an exploratory cohort of undiagnosed UDN cases. SimPheny outperformed state-of-the-art gene prioritization tools and identified numerous high-confidence diagnostic candidates, including diagnoses contemporaneously confirmed by UDN clinical teams and novel diagnoses that had not been previously evaluated. SimPheny is freely available as an open-source web application (SimPheny.iobio; https://simpheny.iobio.io/), and as a standalone tool for cohort-scale analyses (https://github.com/icooperstein/SimPheny).

## Results

### Overview of the SimPheny algorithm for phenotype-first candidate gene prioritization

We developed SimPheny, a computational gene prioritization method that implements a phenotype-first patient matching strategy in cohorts of rare and undiagnosed diseases. SimPheny runs on the hypothesis that patients with similar phenotypes are more likely to share a genetic etiology, and can identify these relationships even when they are not yet documented in existing knowledge bases.

**Figure 1:**
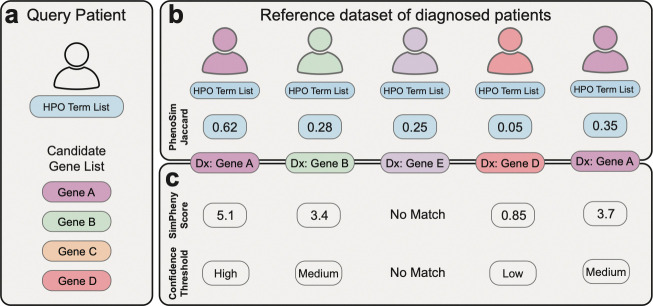
SimPheny prioritizes candidate genes through phenotypic similarity, followed by gene-level evidence from diagnosed reference patients. **a)** SimPheny accepts two inputs for a query patient: (i) a list of HPO terms describing their clinical presentation, and (ii) a candidate gene list. **b)** Phenotypic similarity scores (${\mathrm{PhenoSim}}_{\mathrm{Jaccard}}$) are calculated between the query patient and each patient in a reference dataset of diagnosed patients, based on their respective HPO term lists. **c)** SimPheny identifies overlaps between the query patient’s candidate gene list and the diagnostic genes of each reference patient. Each overlap defines a SimPheny Match, for which a SimPheny Score is calculated by integrating statistical evidence from both the ${\mathrm{PhenoSim}}_{\mathrm{Jaccard}}$ score and the relevance of the contributing gene. Matches are assigned confidence tiers (high, medium, low) based on their SimPheny Score. If no gene overlaps are found, a SimPheny Score cannot be computed (“No Match”). Dx = Diagnosis.

SimPheny accepts two inputs for each query patient: (i) a list of HPO terms describing their clinical features and (ii) a candidate gene list (Fig. 1). It first computes pairwise phenotypic similarity scores, termed ${\mathrm{PhenoSim}}_{\mathrm{Jaccard}}$, by comparing the query patient to individuals in a diagnosed reference cohort. ${\mathrm{PhenoSim}}_{\mathrm{Jaccard}}$ builds on the standard Jaccard index by incorporating both the information content of individual terms and the depth of their shared ancestors within the ontology (Methods).

Next, SimPheny identifies overlaps between the query’s candidate gene list and diagnostic genes in the reference cohort, defining each overlap as a *SimPheny Match*. For each match, it calculates a *SimPheny Score* by integrating the statistical significance of the phenotypic similarity (${\mathrm{PhenoSim}}_{\mathrm{Jaccard}}$) with the relevance of the shared gene (Methods). SimPheny scores are categorized into high-, medium-, or low-confidence tiers, defined through benchmarking, to guide clinical interpretation.

### Phenotypic makeup of the UDN cohort

To develop, benchmark, and apply SimPheny, we used data from the Undiagnosed Diseases Network (UDN), a national research study established to improve the diagnosis and understanding of rare conditions [28]. Our study cohort comprised 2,214 UDN participants with annotated HPO terms, including 767 diagnosed probands or relatives and 1,447 undiagnosed probands (Supplementary Fig. 1, Methods). Of the 767 diagnosed participants, 404 probands had variant data and met the inclusion criteria for the SimPheny benchmarking cohort.

Participants were annotated with a median of 16 HPO terms describing their clinical presentation. The most frequently observed features were general neurological findings, including *global developmental delay* and *seizure* (Supplementary Fig. 2a-b). Nearly half of all participants had phenotypic descriptions classified as disorders affecting the nervous system (Supplementary Fig. 2c; Methods). Spanning a wide range of genetic disorders, both solved and unsolved, this cohort offers a phenotypically diverse foundation for evaluating and applying the SimPheny algorithm.

### Phenotypic similarity captures diagnostic relationships in disease models and patients

To quantify semantic similarity between HPO term sets, we developed ${\mathrm{PhenoSim}}_{\mathrm{Jaccard}}$, a metric that extends the standard Jaccard index. Under this measure, the similarity between two HPO terms is defined as the fraction of information content (IC) – a measure of term specificity based on frequency – contributed by their shared ancestors relative to the total IC of all their ancestors in the HPO (Methods). Scores range from 0 to 1, with 1 indicating an exact match. This approach rewards shared ancestry between terms, particularly when the shared ancestors are highly specific.

**Figure 2:**
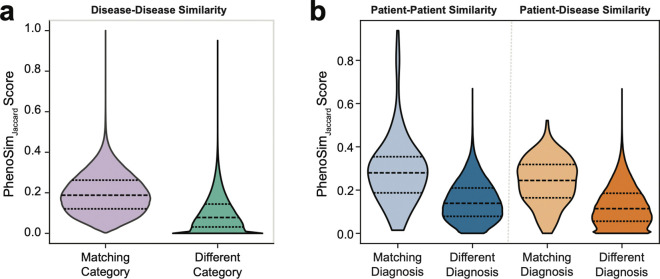
${\mathrm{PhenoSim}}_{\mathrm{Jaccard}}$ measures phenotypic similarity in disease models and patients. **a)** Pairwise ${\mathrm{PhenoSim}}_{\mathrm{Jaccard}}$ scores between 4,120 Orphanet disease models, stratified by whether pairs share the same top-level HPO disease category (*Matching Category*) or belong to different categories (*Different Category*). Dashed lines mark the 25th, 50th, and 75th percentiles. **b)** Subset of 175 diagnosed UDN participants for whom (i) at least one additional UDN participant shared the same causal gene and (ii) an Orphanet disease model was available for that gene. Patient-to-patient ${\mathrm{PhenoSim}}_{\mathrm{Jaccard}}$ scores were stratified by matching versus different genetic diagnoses (blue). Patient-to-disease scores between UDN participants and corresponding Orphanet models were similarly stratified (orange). Dashed lines mark the 25th, 50th, and 75th percentiles.

We first validated ${\mathrm{PhenoSim}}_{\mathrm{Jaccard}}$ using 4,120 disease models from Orphanet [16] (Supplementary Fig. 3). Term-to-term scores were aggregated into set-level similarities using *funSimAvg* [29], which computes the average of the highest-scoring matches from each term in one HPO set to the other, considering both directions of comparison. Pairwise disease-to-disease similarities were calculated and visualized using t-SNE (Supplementary Fig. 4a), revealing that diseases are largely grouped by top-level HPO categories, with distinct groupings for eye, cardiovascular, and other physiological disorders (Supplementary Fig. 4b; Methods). This reflects the expectation that disorders affecting the same system share similar features and thus score higher phenotypic similarity. Quantitatively, disease pairs from the same top-level HPO category had significantly higher ${\mathrm{PhenoSim}}_{\mathrm{Jaccard}}$ scores than pairs from different categories (Fig. 2a).

Although ${\mathrm{PhenoSim}}_{\mathrm{Jaccard}}$ effectively captures relatedness among disease models, curated datasets provide only one model per disease and cannot assess similarity among distinct individuals with the same diagnosis. We therefore evaluated this metric in the UDN cohort, where multiple participants can be diagnosed with the same causal gene.

We evaluated 175 UDN participants for whom both an Orphanet disease model corresponding to their diagnostic gene and at least one additional UDN participant diagnosed in the same gene were available. For each case, we compared patient-to-patient ${\mathrm{PhenoSim}}_{\mathrm{Jaccard}}$ scores with patient-to-disease scores against the corresponding Orphanet disease model. Across the cohort, patient-to-patient similarities were at least as high as patient-to-disease similarities, with comparable separation between matching and non-matching diagnoses (Fig. 2b). These findings show that real patients with the same genetic diagnosis are at least as phenotypically similar to each other as they are to curated disease models. This supports patient-to-patient comparisons as a viable alternative to curated disease models in phenotype-driven diagnostics and highlights the need for multiple diagnosed cases per disease to benchmark SimPheny’s ability to recover shared genetic etiologies.

### Evaluating SimPheny on known diagnoses

We next sought to benchmark SimPheny’s ability to recover known diagnoses from candidate gene lists. Our UDN reference cohort comprised 767 diagnosed UDN participants, including probands and close relatives with HPO-encoded clinical features and at least one definitive diagnostic gene (Supplementary Fig. 1; Methods). We focused on the subset of 557 diagnosed UDN probands who also had genome or exome sequencing data included in the most recent UDN joint callsets. For each of these probands, Exomiser was run on family-level variant data to generate unranked candidate gene lists, which were used as input for SimPheny, thereby mimicking the set of genes an analyst might have considered prior to diagnosis.

Benchmarking SimPheny’s ability to prioritize diagnostic genes requires that each proband’s candidate gene list include their known diagnostic gene. This criterion was met for 404 probands, excluding 153 others (Supplementary Fig. 5) [30]. The final benchmarking cohort comprised 415 diagnostic genes and 29,542 non-diagnostic candidate genes across these 404 probands (Methods). SimPheny was then run for each proband using their HPO terms and candidate gene list, querying against the 767 diagnosed participants in the UDN reference cohort, excluding self-matches to avoid trivial rediscovery (Fig. 1).

A *SimPheny Match* was defined when a candidate gene from a benchmarking proband overlapped the diagnostic gene of a reference participant. For each match, we computed a *SimPheny Score*, which integrates the statistical significance of phenotypic similarity (${\mathrm{PhenoSim}}_{\mathrm{Jaccard}}$) with the cohort-wide frequency of the matched gene (Methods). Matches were labeled as true positives (TPs) if the matched gene was also the diagnostic gene of the benchmarking proband, and false positives (FPs) otherwise. If a candidate gene was not present among the diagnostic genes in the reference cohort, no SimPheny Match could be made, and no score was computed.

Across the 404 benchmarking probands, SimPheny returned 3,442 matches with SimPheny Scores ranging from 0.06 to 6.36. Higher scores reflect both greater phenotypic similarity and stronger statistical evidence that the matched gene and phenotypic overlap is unlikely to occur by chance, given the proband’s HPO terms and candidate gene list. Of the 415 diagnostic genes in the benchmarking cohort, 156 (37.6%) were represented by at least one additional diagnosed case in the reference cohort, allowing the opportunity for a TP match. Each proband had a median of seven scored matches spanning three unique genes (Supplementary Fig. 6), representing a substantial reduction from the median of 41 candidate genes used as input for each proband (Supplementary Fig. 7a). These results demonstrate that SimPheny can prioritize a small, clinically interpretable subset of candidate genes informed by patient phenotype.

**Figure 3:**
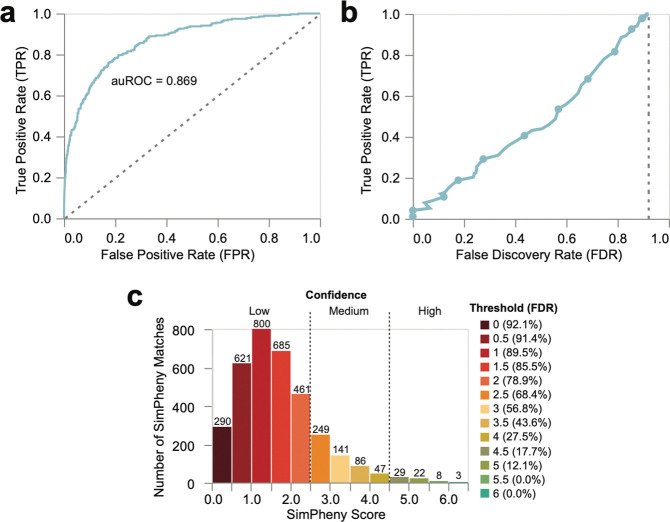
Establishing confidence thresholds for SimPheny matches in the benchmarking cohort. **a)** Receiver Operating Characteristic (ROC) Curve evaluating the ability of SimPheny Scores to distinguish true diagnostic matches from false positives in the UDN benchmarking cohort. The dashed line represents a random classifier. **b)** False Discovery Rate (FDR) curve. Points correspond to SimPheny Score thresholds in 0.5 increments, ranging from 1 (top right) to 6.0 (bottom left). The dashed line represents random expectation, where 92.1% of matches would be predicted to be false due to the class imbalance. **c)** Distribution of SimPheny Scores across all 3,442 matches in the benchmarking cohort, binned in 0.5 increments. Dashed lines mark the three confidence tiers defined by FDR analysis: high (≥ 4.5), medium (2.5–4.5), and low (< 2.5). Numbers above bars indicate the number of matches within each bin. Median SimPheny Score = 1.51.

To define score thresholds for diagnostic confidence, we assessed how well SimPheny Scores discriminated TPs from FPs. Receiver operating characteristic (ROC) analysis yielded an area under the curve (auROC) of 0.869 (Fig. 3a), indicating strong overall performance. In total, 271 matches involved the benchmarking proband’s diagnostic gene (TPs) and 3,171 did not (FPs). As over 90% of matches were FPs, we used the false discovery rate (FDR) as a more informative performance metric in this imbalanced dataset (Methods).

At a permissive threshold (SimPheny Score ≥ 1.5), SimPheny recovered 92.6% of TPs, returning an average of five matches per proband across three genes (Fig. 3b; Supplementary Table 1). However, the FDR at this threshold was 85.5%, indicating that most matches were not diagnostic. At the more stringent threshold SimPheny Score ≥ 4.5, SimPheny recovered 18.8% of TPs, returning on average one match in a single candidate gene, while reducing the FDR to 17.7% – meaning approximately four of every five matches were diagnostic.

Thus, permissive thresholds maximize sensitivity at the cost of precision, whereas stringent thresholds return fewer but higher-confidence candidates (Fig. 3c). We defined three confidence categories: high-confidence (SimPheny Score ≥ 4.5), medium-confidence (2.5 ≤ SimPheny Score < 4.5), and low-confidence (SimPheny Score < 2.5) (Supplementary Table 1). These categories provide interpretable guidance for downstream interpretation, allowing users to balance sensitivity and specificity based on clinical or research context.

### SimPheny outperforms existing candidate gene prioritization tools

Existing candidate gene prioritization tools typically rely on curated gene-phenotype associations from OMIM or Orphanet to rank genes that may explain a patient’s clinical presentation. To benchmark SimPheny against representative approaches, we selected three widely used tools spanning distinct methodological categories: (1) Phrank [31], which scores phenotypic similarity between patient HPO terms and curated gene-phenotype associations; (2) AI-MARRVEL [20], a machine learning framework that integrates gene-phenotype similarity from OMIM with variant-level features (e.g., gnomAD frequency, functional impact) and literature evidence; and (3) Exomiser [18], which combines variant pathogenicity predictions with phenotypic similarity based on curated associations. We evaluated Exomiser under two configurations: the default, which integrates gene-phenotype associations from multiple model organisms, and our previously optimized settings [30], which restrict associations to human data.

We first benchmarked SimPheny’s underlying phenotypic similarity metric (${\mathrm{PhenoSim}}_{\mathrm{Jaccard}}$) against Phrank [31] (Supplementary Methods). ${\mathrm{PhenoSim}}_{\mathrm{Jaccard}}$ identified phenotypically similar patients with shared diagnostic genes more effectively (Supplementary Fig. 8a-b). As Phrank can also score candidate genes by the concordance of their associated phenotypes with a patient’s phenotypes, we compared Phrank gene scores with SimPheny Scores across the 156 probands in our benchmarking cohort who had at least one TP match in the UDN reference dataset. SimPheny markedly outperformed Phrank under both ROC- and FDR-based evaluation (Supplementary Fig. 8c-d), underscoring the advantage of using patient-driven similarity over curated associations to score candidate genes.

We next compared SimPheny with AI-MARRVEL, Phrank, and Exomiser in terms of their ability to prioritize diagnostic genes. As SimPheny can return multiple matches per gene from different diagnosed reference patients (Fig. 1), we ranked candidate genes using a *SimPheny Gene Score* defined as the average of the two highest match scores per gene (Methods). This approach reflects how an analyst might interpret repeated evidence for a gene across multiple patient matches.

**Figure 4:**
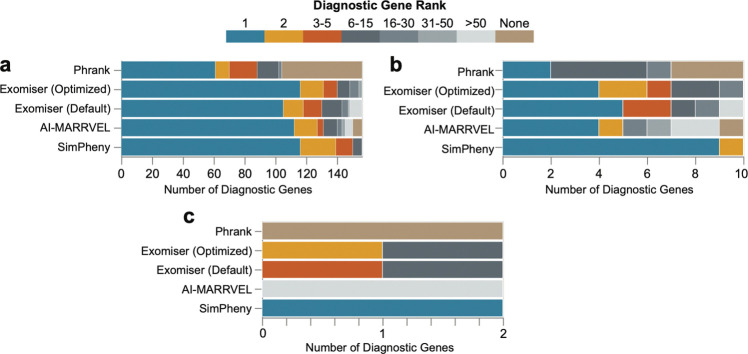
SimPheny outperforms state-of-the-art candidate gene prioritization methods Performance of SimPheny versus Phrank, Exomiser (optimized and default parameters; see Methods), and AI-MARRVEL. Performance was measured as the rank assigned to diagnostic genes by each tool. **a)** Diagnostic genes with at least one true positive match in the UDN reference dataset (n=156) **b)** Diagnostic genes whose associated disease descriptions in OMIM or Orphanet had no direct phenotypic overlap with the proband’s HPO terms (n=10). **c)** Diagnostic genes with *no* documented HPO associations in OMIM or Orphanet (n=2).

SimPheny outperformed all tools in ranking known diagnostic genes, identifying 116 (74.4%) as the top candidate and 150 (96.2%) within the top five. In comparison, AI-MARRVEL ranked 112 (71.8%) as the top candidate and 131 (84.0%) within the top five (Fig. 4a; Supplementary Table 2). Notably, SimPheny never ranked a diagnostic gene below the top 15, in contrast to other tools that extended well past 50 candidates or failed to recover the gene entirely.

Unlike other methods, SimPheny does not rely on curated gene-phenotype associations, enabling it to prioritize emerging or poorly annotated disease genes. To evaluate this advantage, we examined ten diagnostic genes whose associated disease descriptions in OMIM or Orphanet lacked direct phenotypic overlap with the proband’s HPO terms. SimPheny ranked all ten genes within the top two candidates, whereas other tools ranked some beyond the top 50 or failed to rank them at all (Fig. 4b). Including multispecies gene-phenotype association data under Exomiser’s default configuration did not improve ranking performance for genes lacking human-specific phenotype associations. We also identified two probands whose diagnostic genes lacked *any* associated HPO terms. SimPheny ranked both as the top candidate, while AI-MARRVEL ranked them below 50th, and Phrank could not score them, as it can only evaluate genes annotated in OMIM or Orphanet (Fig. 4c).

Collectively, these results demonstrate that SimPheny’s phenotype-first patient matching approach enables superior prioritization of diagnostic genes, especially in cases where curated associations are sparse, absent, or incomplete. Its independence from curated gene-phenotype knowledge bases is a key strength, allowing SimPheny to prioritize candidates that other frameworks cannot. Even in well-established disease contexts, where existing frameworks typically identify the diagnostic gene, SimPheny achieves superior diagnostic gene ranking (Fig. 4a).

### Using SimPheny to diagnose previously unsolved UDN probands

**Figure 5:**
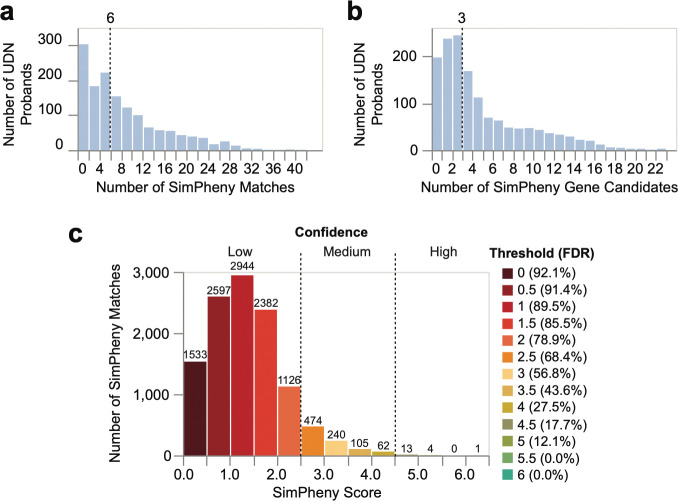
Applying SimPheny to the UDN discovery cohort prioritizes candidate genes for diagnostic review. **a)** Number of SimPheny Matches returned per proband when querying 1,447 undiagnosed probands against 767 diagnosed UDN reference participants. A single proband may have multiple matches to the same gene if multiple reference cases are diagnosed in that gene. The dashed line indicates the median number of matches per proband. 197 probands (13.6%) had no matches. **b)** Number of unique SimPheny gene candidates returned per proband. The dashed line indicates the median number of candidate genes per proband. **c)** Distribution of SimPheny Scores across 11,481 matches in the discovery cohort, binned in 0.5 increments. Dashed lines mark confidence tiers defined by FDR benchmarking analyses (Fig. 3; Supplementary Table 2): high (≥ 4.5), medium (2.5–4.5), and low (<2.5). Numbers above bars indicate counts per bin. Median SimPheny Score = 1.27. The 18 high-confidence matches correspond to the 18 SimPheny candidates prioritized for diagnostic review.

Having benchmarked SimPheny on known diagnoses, we next applied it to 1,447 undiagnosed probands in our discovery cohort, querying against the 767 diagnosed UDN participants in the reference dataset (Fig. 1; Supplementary Fig. 1). Using Exomiser-derived candidate gene lists and HPO-encoded clinical features as input, SimPheny identified and scored 11,481 candidate gene matches, with scores ranging from 0.03 to 6.09. Probands had a median of six matches spanning three unique candidate genes – a substantial reduction from the 42-gene median in the original candidate lists (Fig. 5; Supplementary Fig. 7b).

From our benchmarking analyses, we defined high-confidence SimPheny matches as those with SimPheny Score ≥ 4.5, corresponding to an estimated FDR of 17.7%, or roughly four true diagnostic matches for every five returned (Table 1; Fig. 3). We prioritized the 18 high-confidence candidate matches from the discovery cohort for diagnostic review, including evaluation of phenotypic concordance with the matched reference participant, inheritance patterns, variant pathogenicity predictions, and other supporting clinical features (Table 2; extended in Supplementary Table 3).

Among the 18 high-confidence matches, eight (44.4%) were subsequently determined to represent confirmed diagnoses upon diagnostic review. Five of these matches corresponded to diagnoses that UDN clinical teams had independently reached but were not yet entered into the internal UDN database at the time of our cohort curation; we refer to these as *contemporaneously diagnosed* candidates. One match aligned with a tentative diagnosis that was excluded from our diagnosed cohort due to diagnostic uncertainty (Methods).

*RPL13* was ranked as the top candidate in a proband with two high-confidence matches and a third just below the threshold (SimPheny Score = 4.45). This proband was evaluated by the UDN five years prior, resulting in a report of unclassified spondyloepimetaphyseal dysplasia with no identified genetic cause. Communication with the UDN clinical site and the original reporting laboratory revealed that the family’s genome sequencing had been analyzed and reported before publication of the study implicating *RPL13* in Spondyloepimetaphyseal dysplasia, Isidor-Toutain type [32]. Notably, this UDN family’s *RPL13* variant was included in that initial report and has been published again since [33], establishing it as a confirmed disease-causing variant. Prompted by SimPheny’s nomination, segregating testing confirmed the pathogenic variant in the proband and four affected relatives, while the unaffected sibling tested negative. This clinical and molecular explanation has now been returned to the family as a certain diagnosis, underscoring SimPheny’s utility in reanalysis for identifying candidates with emerging gene-phenotype-disease relationships.

Of the remaining ten matches, one was explicitly rejected by the clinical team overseeing the case, while the other nine remain unresolved pending further evaluation. Some of these candidate genes represent variants classified as benign or likely benign in ClinVar [34] that are likely to be rejected, yet others represent candidate genes with variants of uncertain significance or conflicting interpretations (Supplementary Table 3).

These matches demonstrate SimPheny’s clinical utility in generating actionable diagnostic leads and surfacing missed candidate genes in patients unresolved by standard analyses.

### Integrating expanded diagnosed reference cohorts enhances SimPheny’s diagnostic reach

SimPheny’s diagnostic power is inherently constrained by the scope of its diagnosed reference cohort: a true diagnostic match cannot be identified if no reference patient has been diagnosed in the relevant gene. Using the UDN-only reference cohort, SimPheny recovered 156 diagnostic genes, accounting for 37.6% of the 415 diagnostic genes in our benchmarking set. To assess whether expanding the reference cohort could improve recovery without compromising performance, we incorporated three additional resources: 6,521 individuals from the Phenopacket Store [35], 11,797 ClinVar “pseudopatients” extracted from variant-level submissions (excluding submissions from the UDN) [34], and 4,576 patients from DECIPHER [36] (Supplementary Methods).

**Figure 6:**
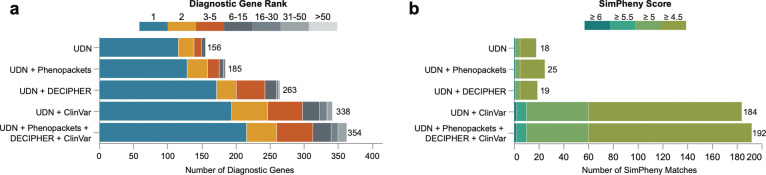
Expanding the diagnosed reference cohort increases recovery of diagnostic genes and high-confidence SimPheny candidates. **a)** SimPheny performance ranking known diagnostic genes as the diagnosed reference cohort expands. Numbers indicate the total number of diagnostic genes recovered (out of 415) for each cohort. **b)** Number of high-confidence SimPheny matches (SimPheny Score ≥ 4.5) identified in the discovery cohort as the reference cohort expands. Numbers reflect the total count of high-confidence matches identified for each reference dataset.

Expanding the reference cohort increased the number of represented genes from 592 in the UDN-only set to 2,939 across all four combined datasets. Each resource contributed additional gene coverage, and integration of all four enabled recovery of 354 diagnostic genes (85.3%) from the benchmarking cohort (Fig. 6a; Supplementary Table 4). Although the total number of SimPheny matches and candidate genes per proband increased (Supplementary Fig. 9), the ranking performance remained robust, with 88.2% to 96.2% of diagnostic genes still ranked within the top five SimPheny candidates, depending on the expanded cohort used (Fig. 6a).

Across the 404 benchmarking probands, SimPheny returned 98,423 matches against the combined reference dataset, including 7,869 involving the proband’s diagnostic gene (TPs). Critically, precision was maintained despite the expanded and more heterogeneous reference set: at the high-confidence threshold of SimPheny Score ≥ 4.5, the FDR remained comparable to the UDN-only analysis (16.3% versus 17.7%; Supplementary Table 5; Table 1; Supplementary Fig. 10). This stability in FDR indicates that manual review burden does not increase, and high-confidence matches remain similarly enriched for TPs. The primary impact of expanding the reference cohort was an increase in lower-scoring TP matches, resulting in a lower proportion of TPs exceeding the high-confidence threshold (9.4% versus 18.8% TPR). These results demonstrate that SimPheny scales effectively with heterogeneous, multi-source reference cohorts, preserving precision while substantially broadening the scope of diagnosable genes.

We next applied the expanded reference cohort to the 1,447 undiagnosed probands in the discovery cohort, yielding 309,834 scored matches (Supplementary Table 5). Thirteen of the 17 probands prioritized for manual review gained additional matches in the same gene, including five of the seven ultimately confirmed diagnoses (Supplementary Table 6). The number of high-confidence SimPheny matches (SimPheny Score ≥ 4.5) rose more than tenfold - from 18 with the UDN-only reference to 192 using the combined cohort (Fig. 6b; Supplementary Fig. 11).

Together, these findings show that a large, comprehensive, and diverse diagnosed reference patient cohort enhances SimPheny’s ability to recover diagnostic genes, strengthen evidence for candidate matches, and support diagnoses in previously unsolved cases. These results underscore the importance of building and integrating comprehensive, well-phenotyped reference resources to enable scalable, phenotype-driven diagnostics for rare diseases.

### Offering SimPheny via a visual, real-time analysis interface with SimPheny.iobio

To facilitate real-time exploration of patient similarity and candidate gene prioritization, we developed *SimPheny.iobio*, an open-source web application available at https://simpheny.iobio.io/ and source code at https://github.com/iobio/SimPheny.iobio. Users can input a list of HPO terms and, optionally, a candidate gene list, and query against a selected diagnosed reference cohort (UDN, ClinVar, Orphanet, DECIPHER, or the Phenopacket Store).

When a candidate gene list is provided, SimPheny.iobio computes SimPheny Scores between the user’s candidate genes and matched reference patients, ranking returned genes in an interactive side panel. If no gene list is provided, the tool supports phenotype-only queries by computing ${\mathrm{PhenoSim}}_{\mathrm{Jaccard}}$ scores to identify phenotypically similar diagnosed individuals and their associated diagnostic genes. In this mode, SimPheny Scores are not calculated as they require gene-level input. To support phenotype-only use cases, we provide benchmarking of ${\mathrm{PhenoSim}}_{\mathrm{Jaccard}}$ thresholds in the Supplementary Materials (Supplementary Fig. 12).

This interactive platform enables the real-time exploration of patient similarity and candidate genes, facilitating the adoption of SimPheny in both research and clinical settings.

## Discussion

We present SimPheny, a phenotype-first framework for gene prioritization that leverages phenotypic similarity between patients. In contrast to variant-first pipelines, which begin with variant filtering and apply phenotype data secondarily, SimPheny starts with patient-to-patient phenotypic overlap and then integrates genotypic evidence to generate diagnostic hypotheses. This approach enables recovery of shared genetic etiologies in the absence of curated gene-phenotype associations.

Across benchmarking analyses, SimPheny consistently outperformed existing tools, particularly for causal genes lacking curated associations (Fig. 4; Supplementary Table 2). Whereas Phrank, Exomiser, and AI-MARRVEL rely on OMIM and Orphanet annotations to connect variants to phenotypes, SimPheny bypasses this dependency by using patient-to-patient comparisons. For genes with limited or absent phenotype associations, SimPheny always ranked the diagnostic gene among the top two candidates, whereas other tools often placed them beyond the 50th or failed to recover them altogether.

SimPheny’s clinical utility was illustrated through case-level analyses. Among the 18 high-confidence matches prioritized for review in our discovery cohort, eight (44.4%) corresponded to confirmed diagnoses (Table 2). Five of these candidates were contemporaneously identified by UDN clinical teams, yet the matches surfaced by SimPheny highlight its value in prioritizing and confirming diagnostic leads.

In one proband with unexplained osteogenesis imperfecta (OI), Exomiser deprioritized *KIF5B* due to the absence of associations linking this gene to the proband’s phenotypes (Supplementary Table 3). In contrast, SimPheny returned *KIF5B* as the top gene candidate by matching the proband to a case diagnosed with *KIF5B-related OI*, demonstrating SimPheny’s ability to recover diagnostic genes that variant-first approaches miss or rank poorly due to gaps in association knowledge.

In another proband with unclassified spondyloepimetaphyseal dysplasia, SimPheny prioritized *RPL13* as the top candidate, supported by multiple high-confidence matches. Although *RPL13* had not been flagged during the initial UDN evaluation, subsequent studies established this family’s *RPL13* variant as the cause of Spondyloepimetaphyseal dysplasia, Isidor-Toutain type [32, 33]. SimPheny’s nomination of the family’s pathogenic *RPL13* variant enabled the clinical team to return a definitive diagnosis to this family after five years, highlighting SimPheny’s capacity to surface clinically meaningful diagnostic leads and support reanalysis efforts for emerging gene-disease relationships.

SimPheny’s performance depends on the scope of its diagnosed reference dataset: a gene must be represented to be recoverable. Expanding the reference to include UDN, ClinVar, the Phenopacket Store, and DECIPHER cases increased gene coverage fivefold (from 592 to 2,939 genes) and diagnostic gene recovery from 37.6% to 85.3% compared to the UDN-only reference (Supplementary Table 4). Despite variation in phenotyping quality among external sources (Supplementary Methods), SimPheny maintained strong ranking performance, recovering 88.6% of diagnostic genes within the top five candidates (Fig. 6a). Expansion also increased the number of high-confidence SimPheny matches in the discovery cohort by more than tenfold (Fig. 6b), and external matches provided supporting evidence in cases that underwent clinical review (Supplementary Table 6). Together, these findings demonstrate that large, diverse, and variably curated reference datasets can be leveraged for phenotype-driven diagnostics and highlight SimPheny’s generalizability to real-world clinical cohorts.

To support broader adoption, we developed *SimPheny.iobio*, a real-time web interface that enables users to enter HPO terms and candidate genes and explore patient similarity across multiple reference cohorts. This platform lowers technical barriers and facilitates scalable applications across diagnostic and research settings.

While this study focused on matching undiagnosed cases to diagnosed references, thereby excluding the chance for novel disease discovery, SimPheny nevertheless recovered high-confidence matches in genes with limited prior phenotypic evidence, highlighting its potential in the early stages of disease gene characterization. Ongoing work extends SimPheny to undiagnosed-to-undiagnosed patient matching, enabling identification of patients with shared phenotypes and variants in the same genes, independent of existing association evidence. Future directions include evaluating SimPheny’s potential to resolve multigenic diagnoses, where multiple high-confidence gene matches contribute to a proband’s presentation.

Although the UDN represents a particularly challenging and diagnostically complex population, its deep phenotyping likely enhances SimPheny’s accuracy. However, SimPheny’s strong performance across heterogeneous reference datasets with limited or noisy phenotypic data suggests its broad utility across diverse clinical contexts. Moreover, as many solved UDN cases involve well-established gene-phenotype associations, benchmarking likely underestimates SimPheny’s advantage in scenarios where such knowledge is sparse or absent.

SimPheny offers a scalable, phenotype-first framework for gene prioritization in rare disease diagnostics. By leveraging patient-to-patient phenotypic similarity, it bypasses key limitations of variant-first tools and operates independently of curated gene-phenotype-disease associations. As newly diagnosed patients enter the reference cohort, SimPheny can immediately surface matches in similar cases, supporting reanalysis and early recognition of emerging gene-disease relationships. Rather than relying on periodic updates to OMIM or Orphanet, SimPheny can help generate the case-level evidence that ultimately informs these databases. Its successful application to previously unsolved UDN cases illustrates how phenotype-driven matching can recover diagnoses and nominate candidates before associations are formally documented, allowing detection as knowledge evolves and new conditions emerge. In this way, SimPheny directly addresses gaps left by association-dependent tools and advances rare disease diagnostics.

## Methods

### Overview of the analytical workflow

Our study followed a multistep workflow to integrate genomic and phenotypic data from the Undiagnosed Diseases Network (UDN) and evaluate the performance of the SimPheny framework (Fig. 1). First, we harmonized exome and genome sequencing data across the UDN cohort to ensure consistent variant representation. We then assembled and curated UDN participant phenotype data, removing uninformative features to improve downstream similarity analyses. Candidate gene lists were then generated using Exomiser and, together with patient phenotypes, used as inputs to SimPheny. SimPheny computes patient-to-patient phenotypic similarity and identifies overlapping candidate genes with diagnosed reference cases, assigning each match a statistical score. We benchmarked SimPheny using a cohort of diagnosed UDN probands to establish confidence thresholds and then applied this framework to undiagnosed probands to identify candidate diagnostic genes.

### Harmonization of exome and genome sequencing data

Cohort-level WGS and WES variant datasets from UDN participants were generated to provide consistent variant representation across all cases. For WGS, unaligned, paired-end FASTQ files from 5,412 unique samples corresponding to 5,353 individuals from 1,772 families enrolled in the UDN as of November 2023 were aligned to human reference hg38 (with decoys and all alt contigs) and processed to produce per-sample GVCF files in the Amazon Web Services (AWS) cloud using the Clinical Genome Analysis Pipeline (CGAP, https://cgap.hms.harvard.edu/). Per-sample GVCFs were then egressed to the local Harvard institutional cluster, where single nucleotide variants (SNVs) and short insertions/deletions (indels) were jointly called across all samples using Sentieon. For WES, unaligned FASTQ pairs from 1,454 unique samples corresponding to 1,439 individuals from 314 families enrolled in the UDN as of June 2024 were processed using the same workflow. All processing steps were performed in parallel on the Harvard institutional cluster [37]. From these cohort-level variant datasets, we extracted multisample VCF files [38] for each UDN case, including affected probands and relevant affected and unaffected relatives.

### Phenotype curation and UDN data assembly

#### UDN cohort assembly and diagnostic categorization

We identified 2,698 UDN participants comprising probands and affected family members with HPO-encoded phenotype lists recorded during their clinical evaluations in reports retrieved from the internal UDN database on January 10, 2025. Each UDN diagnosis is assigned one of four certainty levels: *certain, highly likely, tentative,* or *low.* A *certain* diagnosis represents a confident genetic finding with minimal uncertainty, while a *highly likely* diagnosis indicates some uncertainty but is considered sufficient for clinical decision-making [28]. For cases with multigenic diagnoses, each gene-condition pair is assigned its own certainty level.

For this study, participants were considered **diagnosed** if they had *certain* or *highly likely* genetic diagnoses, and **undiagnosed** if they had no diagnosis, *tentative* or *low* certainty diagnoses, or clinical-only (i.e., lacking causal genetic variants) diagnoses. We only retained diagnoses with single gene findings (i.e., excluded diagnoses in large structural variants encompassing multiple genes). Based on this criteria, 767 participants were classified as diagnosed (731 probands and 36 relatives), and 1,931 were classified as undiagnosed. Relatives were retained in the diagnosed dataset if their phenotype sets did not exactly match their related proband. The 767 diagnosed participants, representing 592 unique genes, comprised our **UDN reference cohort** (Supplementary Fig. 1). 462 genes were implicated in a single participant, and 130 in more than one.

#### Phenotype curation

Phenotype annotations for UDN participants, Orphanet disease descriptions, and external reference datasets (ClinVar, the Phenopacket Store, and DECIPHER; Supplementary Methods) were curated to exclude terms related to prenatal and perinatal history. These terms were commonly pre-populated in earlier UDN data collection interfaces, resulting in their frequent inclusion in participant records, regardless of clinical relevance. Prenatal and perinatal history HPO terms do not usually describe the phenotypes of the genetic disorder under investigation. As such, these terms contribute noise to our phenotype-first analysis and so were removed from all participant and disease descriptions in this study. A complete list of removed terms is provided in the supplementary materials.

#### Disease category assignment

We defined 23 disease categories corresponding to the first-level child terms of *Phenotypic abnormality* in the HPO. This “top-level” of the HPO includes broad terms such as *Abnormality of the nervous system* and *Abnormality of the eye*, which serve as parent categories for more specific HPO terms. Due to the hierarchical structure of the HPO, any more specific term must trace to one or more of these top-level terms as an ancestor. To assign each patient to a single disease category, we initialized a score of 0 for each of the 23 top-level terms. For an HPO term set describing a patient or disease, each HPO term was mapped to its top-level ancestors, and the term’s information content (IC, derived from OMIM frequencies) was added to the score of each corresponding top-level ancestor. This approach weights rarer, more informative terms and therefore their top-level ancestor more heavily than common, nonspecific terms. The top-level term with the highest cumulative IC-based score was then assigned as the term set’s primary disease category.

### Candidate gene list generation with Exomiser

#### Input files and filtering

Standard Exomiser inputs include a multisample family VCF file [38], a corresponding pedigree file in PED format, and proband phenotype annotations in HPO format [39]. For each UDN family, multisample VCFs including probands and relatives were extracted from the harmonized WGS or WES variant datasets. VCFs were filtered to exclude variants with an alternate allele of “*”, genotype quality (GQ) < 20, or heterozygous calls with a variant allele frequency (VAF) less than 0.15 or greater than 0.85 [40, 41]. Proband phenotype terms were obtained from the UDN internal database, with prenatal and perinatal HPO terms removed as described above. Pedigree files were obtained from the cohort-level WGS or WES datasets.

#### Optimized versus default parameterization

We previously identified optimized parameter settings for running Exomiser on the UDN cohort [30]. To generate candidate gene lists for SimPheny, we ran Exomiser v14.0.0 with dataset version 2406, using the optimized parameters (an example YML file is provided on GitHub). Briefly, optimization included restricting the hiPHIVE phenotype prioritization algorithm [42] to human-only associations and incorporating REVEL [43], MVP [44], AlphaMissense [45], and SpliceAI [46] as variant pathogenicity predictors. The maximum allele frequency filter was set to 0.02 to accommodate compound heterozygous diagnoses, which often involve genetic variants with higher allele frequencies.

For benchmarking analyses, we also ran Exomiser using default settings, under which hiPHIVE integrates human, mouse, zebrafish, and protein-protein interaction (PPI) network data, and variant pathogenicity is evaluated using only REVEL and MVP.

Exomiser calculates gene-level variant and phenotype scores, which are combined to generate ranked lists of protein-coding candidate genes under multiple modes of inheritance [47]. For downstream SimPheny analyses, we **retained only genes with either a variant *or* phenotype score greater than 0.5, ensuring that variants with strong pathogenicity predictions but weak phenotypic evidence were not excluded**. No additional filtering (i.e., based on Exomier-derived p-values or combined scores) was applied.

Critically, SimPheny’s performance depends on the candidate gene list it receives – it cannot prioritize an absent gene. Thus, the effectiveness of phenotype-first matching relies on the quality and breadth of upstream variant filtering. To account for this dependency, we deliberately retained not only top-scoring Exomiser candidates, but also genes harboring variants with strong pathogenicity predictions that were otherwise deprioritized due to limited or absent phenotypic evidence. This approach ensured that SimPheny could evaluate candidates that variant-first tools might have overlooked.

### Curation of benchmarking and discovery cohorts

#### Curation of the SimPheny benchmarking cohort

We restricted the benchmarking cohort to probands, as candidate gene lists were constructed using all available family-level variant data. Of the 767 diagnosed UDN participants in the UDN reference cohort, 557 were probands with variant data in the harmonized WES or WGS variant datasets, representing 579 diagnostic genes (19 probands had more than one genetic diagnosis). For probands with both WES and WGS data (n=26), WGS results were used.

Exomiser was run on each proband’s family-level VCF using curated HPO term lists and available pedigrees under optimized parameters. Genes with either a variant *or* phenotype score greater than 0.5 were retained, and the remaining list of ranked genes was assessed for presence or absence of the proband’s diagnostic gene(s), regardless of the specific variant or inheritance model contributing to that gene’s score. Of the 579 diagnostic genes, 415 (71.7%) were prioritized at any rank. The remaining 164 genes were not prioritized due to primarily dataset or algorithmic limitations: 33% (n=54) involved structural variants, which are not represented in the jointly called datasets, 30% (n=50) involved noncoding variants, which are excluded by Exomiser’s protein-coding focus, and 22% (n=36) were missed due to pedigree inaccuracies (e.g., relatives misclassified as unaffected despite mild phenotypes) (Supplementary Fig. 5). Probands whose diagnostic genes were absent from the Exomiser output were excluded, as SimPheny cannot recover genes not included in input candidate lists. We note that this exclusion reflects a limitation of the input data rather than of SimPheny itself.

After these exclusions, the final **SimPheny benchmarking cohort** comprised 404 probands with 415 diagnostic genes (Supplementary Fig. 13a). Each proband was assigned an unranked candidate gene list using the filtered Exomiser results to use as input for SimPheny, mirroring the candidate gene sets available for undiagnosed patients in real diagnostic workflows (Fig. 1). Candidate gene lists contained a median of 103 genes before filtering, and 41 after applying the 0.5 variant/phenotype gene score requirement (Supplementary Fig. 7a).

#### Curation of the SimPheny discovery cohort

To evaluate SimPheny in a translational context, we assembled a discovery cohort of undiagnosed UDN probands with available variant data. Of the 1,931 undiagnosed participants, 1,447 probands had WGS or WES data present in the harmonized, jointly called datasets across various family structures (Supplementary Fig. 1; Supplementary Fig. 13b). For probands with both WGS and WES data (n=108), we used the WGS results.

Exomiser was run on each proband’s family-level VCF with curated HPO phenotype lists using optimized parameters. Each proband’s candidate list contained a median of 119 unique genes prior to filtering. After retaining only genes with either a variant or phenotype score greater than 0.5, the candidate lists contained a median of 42 genes (Supplementary Fig. 7b). These filtered lists were used as candidate gene lists to run SimPheny.

### SimPheny framework

#### Phenotypic similarity metric (${\mathrm{PhenoSim}}_{\mathrm{Jaccard}}$)

To quantify the similarity between HPO term sets, we developed ${\mathrm{PhenoSim}}_{\mathrm{Jaccard}}$, a modified Jaccard index that incorporates the information content (IC) of HPO terms.

For two HPO terms *T*_*1*_ and *T*_*2*_, their phenotypic similarity is defined as the intersection of their ancestral terms divided by the union of their ancestral terms:

$${PhenoSim}_{\mathrm{Jaccard}}(T1,\;T2)=\frac{intersection(T1,\;T2)}{union(T1,\;T2)}\;\;=\frac{\sum \;\;IC_{\mathrm{CommonAncestors}}(T1,\;T2)}{\sum \;\;IC_{\mathrm{Allancestors}}(T1,\;T2)}$$

The intersection is defined as the sum of IC values for all ancestors shared by *T*_*1*_ and *T*_*2*_ (including the terms themselves), while the union is the sum of IC values across all ancestors of both terms. Scores range from 0 to 1, with 1 representing exact matches. Unlike the classic Jaccard index, which considers only set overlap, ${\mathrm{PhenoSim}}_{\mathrm{Jaccard}}$ rewards shared ancestry between terms in the ontology, particularly when those ancestors are highly specific, represented by high IC values.

The IC of a term T is defined as:

$$IC=-log\left(T_{freq}\right)$$

Where $T_{freq}$ is the proportion of OMIM diseases annotated with the term. IC values were computed using the pyHPO python package [48], and term-to-term similarities were aggregated into set-to-set similarities using the *funSimAvg* method implemented in this package [29]. Briefly, *funSimAvg* computes the similarity between two HPO term sets by averaging, for each term in one set, the highest semantic similarity it achieves with any term in the other set.

### Definition of SimPheny Matches

For each query proband, SimPheny identifies matches as overlaps between the proband’s Exomiser-derived candidate gene list and the diagnostic genes of reference patients, supported by phenotypic similarity between the two individuals. Each such overlap defines a **SimPheny Match** (Fig. 1). Self-matches were excluded when the UDN cohort was used as the reference dataset to avoid trivial rediscovery by a proband matching with themself.

A single gene could yield multiple matches if several reference cases are diagnosed in that gene. Each match is scored independently based on the phenotypic similarity to the corresponding reference case, and these gene-level matches were later aggregated into SimPheny Gene Scores for downstream ranking (see below). Conversely, diagnostic genes present in only a single UDN individual cannot be validated in benchmarking with the UDN reference cohort, as no independent reference case exists for comparison.

### Calculation of the SimPheny Score

To quantify the significance of each SimPheny match, we calculated two empirical p-values: (1) *pheno p*, the probability that the observed phenotypic similarity score (${\mathrm{PhenoSim}}_{\mathrm{Jaccard}}$) between a query and reference case could occur by chance, and (2) *gene p*, the probability that the matched gene would appear in the query’s Exomiser-derived candidate list by chance based on cohort-wide expectations. These two measures were then combined into the *SimPheny Score*, which jointly captures phenotypic concordance and gene-level specificity.

### Phenotypic similarity significance (*pheno p*)

To generate a null distribution for phenotypic similarity, we performed 10,000 Monte Carlo simulations per query-reference SimPheny match. In each simulation, a randomized phenotype list was generated for the query proband by sampling without replacement from the full corpus of HPO annotations in the reference dataset (42,575 annotations across 5,149 unique terms in the UDN). Sampling was weighted by term frequency to preserve population-level biases. Each simulated list was capped at 10 terms to reduce computational burden, but matched the original list length if shorter. Each simulated term list was then compared to the reference’s observed term list using the ${\mathrm{PhenoSim}}_{\mathrm{Jaccard}}$ metric.

The *pheno p* was defined as:

$$pheno\;p=\frac{1\;+\;\#\left\{{\mathrm{PhenoSim}}_{\mathrm{Simulated}}\;\geq \;{\mathrm{PhenoSim}}_{\mathrm{Observed}}\right\}}{1\;+\;N}$$

Where N = 10,000 simulations. The +1 correction prevents zero-valued p-values.

Although Monte Carlo sampling assumes independence between HPO terms, the hierarchical structure of the HPO introduces correlations. As a result, some simulated lists may partially capture the query patient’s true phenotypic presentation, potentially skewing p-values despite incorrect or overly general terms.

### Gene match specificity (*gene p*)

To account for genes that are frequently prioritized by Exomiser – such as large genes or those associated with many nonspecific phenotypes – we generated an empirical null distribution to model the expected frequency with which each gene appears in candidate gene lists.

We compiled a corpus of all genes returned by Exomiser across 1,851 UDN probands (404 diagnosed and 1,447 undiagnosed), retaining duplicate gene entries to preserve the empirical frequency distribution. For probands with both WES and WGS data, only WGS results were used. For each query-to-reference match, we simulated 10,000 gene lists by sampling with replacement from this corpus. The number of genes in each simulated list matched the length of the proband’s filtered Exomiser-derived candidate gene list (genes with variant or phenotype score greater than 0.5).

The genep was defined as:

$$\mathrm{gene}\;p=\frac{1\;+\;\#\{\mathrm{simulated}\;\mathrm{lists}\;\mathrm{containing}\;\mathrm{matched}\;\mathrm{gene}\}}{1\;+\;N}$$

Where N = 10,000 simulations. The presence or absence of the gene was recorded for each simulated list, regardless of how many times it appeared in the sampled list. Genes can be returned by Exomiser multiple times (e.g., different modes of inheritance), but for simplicity and interpretability, we reduced this to a binary presence/absence.

This sampling approach reflects the natural frequency distribution of genes prioritized by Exomiser across the UDN cohort. Genes frequency prioritized appear more often in the corpus and thus are more likely to appear in the simulated lists. This strategy provides an empirically calibrated null model that adjusts for biases inherent to the tool, including effects of gene size, annotation density, and other Exomiser-specific biases.

### Unifying match significance using Empirical Brown’s Method

To generate a unified measure of statistical support for each SimPheny match, we combined the *pheno p* and *gene p* into a single **combined p-value** using Empirical Brown’s Method (EBM) [49]. EBM extends Fisher’s method [50] for combining p-values by incorporating the empirical covariance structure of the input p-values, allowing for accurate p-value combination even when the input statistics are not independent.

This adjustment is critical, as *pheno p* and gene *p* are expected to be correlated: higher phenotypic similarity (*pheno p*) may increase the likelihood that a diagnostic gene appears in the Exomiser candidate gene list (*gene p*), as similar or matching phenotypes have overlapping gene-phenotype associations used by Exomiser to score and rank genes.

Unlike Fisher’s method, which assumes independence, and Stouffer’s method [51], which requires known z-scores and correlation coefficients, EBM uses empirical estimates of correlation derived from the data and outputs a single interpretable p-value per SimPheny match. The ***combined p*** resulting from EBM represents the joint probability of observing a SimPheny match under the null model, accounting for the dependency between ***pheno p*** and ***gene p***.

To parameterize EBM, we used SimPheny matches generated from the benchmarking cohort of 404 diagnosed UDN probands against each of four reference datasets: UDN, ClinVar, the Phenopacket Store, and DECIPHER. For each reference dataset, we transformed *pheno p* and *gene p* values into z-scores using the inverse normal transformation, estimated the empirical correlation (ρ) between them, and derived dataset-specific scaling factors and degrees of freedom. These fixed parameters (Supplementary Table 7) were then used to calculate combined p-values for all subsequent analyses of undiagnosed probands using SimPheny queried against the appropriate reference dataset.

Each reference dataset required its own fixed EBM parameters, as the distributional properties and dependencies between *pheno p* and *gene p* vary with the structure and content of the reference cohort. For example, the UDN cohort is enriched for neurological phenotypes, and participants are more deeply phenotyped compared to ClinVar pseudopatients. Using dataset-specific calibrations ensures that SimPheny Scores are appropriately scaled and comparable across different analysis contexts. Applying a single set of EBM parameters across heterogeneous datasets would distort the null distribution and compromise the interpretability of match significance.

### SimPheny Score definition and calibration

To provide an interpretable and generalizable measure of match significance, we defined the **SimPheny Score** as the negative ${\text{log}}_{10}$ of the EBM combined p-value:

$$SimPheny\;Score\;=-{log}_{10}(combined\;p)$$

This score increases with stronger joint evidence of phenotypic similarity and gene-level specificity. A high SimPheny Score reflects a rare combination of both a phenotypically similar match and a gene that is unlikely to appear by chance in the candidate gene list.

Although multiple-testing-adjusted p-values using the Benjamini-Hochberg false discovery rate (FDR) correction [52] are also reported for completeness, these values are not suitable for generalization across cohorts. Adjusted p-values depend on the number of comparisons in a given dataset and are therefore cohort-specific. In contrast, the SimPheny score is independent of dataset size and composition, making it a portable metric that can be applied to new patients without requiring recalibration.

To define meaningful confidence thresholds for the SimPheny Score, we benchmarked performance across a range of scores using the diagnosed UDN benchmarking cohort. These benchmarks were used to classify matches into high-, medium-, and low-confidence categories, as described in the Results.

### Ranking candidate genes using the SimPheny Gene Score

A single query proband can generate multiple SimPheny matches to the same gene via different reference patients. To aggregate these matches into a gene-level score for candidate gene ranking, we defined the **SimPheny Gene Score** as the average of the two highest SimPheny Scores observed for matches in that gene. This approach preserves evidence from multiple supporting matches while preventing numerous low-confidence matches from disproportionately affecting the gene score. If only a single match was observed for a given gene, the SimPheny Gene Score was set equal to that SimPheny Score.

Each proband’s candidate genes were then ranked in descending order by their SimPheny Gene Scores, producing a prioritized gene list. This ranked list was used for comparative evaluation with other prioritization tools. SimPheny Gene Scores thus provide a scalable and interpretable framework for gene prioritization that integrates multiple layers of patient similarity evidence.

### Benchmarking design

The curated benchmarking cohort of 404 diagnosed UDN probands was used as a truth set to evaluate SimPheny’s ability to recover known diagnostic genes. For each proband, we ran SimPheny on the Exomiser-derived candidate gene lists (filtered for variant or phenotype score greater than 0.5) and the proband’s HPO-encoded clinical features, querying against a reference dataset of diagnosed individuals (Fig. 1). Each match was classified as a true positive (TP) if the candidate gene corresponded to the proband’s known diagnostic gene, or a false positive (FP) otherwise. A SimPheny score was computed for each match as described above.

We assessed performance across a range of SimPheny Score thresholds. At each threshold (e.g., all matches with SimPheny Score ≥ 1), we calculated the following metrics:

$$True\;Positive\;Rate\;(TPR)=\frac{TP}{TP\;+\;FN}=Sensitivity$$

$$False\;Positive\;Rate\;\left(FPR\right)=\frac{FP}{FP\;+\;TN}=\left(1-Specificity\right)$$

$$False\;Discovery\;Rate\;\left(FDR\right)=\frac{FP}{FP\;+\;TP}=\left(1-Precision\right)$$

Where:

TP = number of true positive matches with scores ≥ threshold

FP = number of false positive matches with score ≥ threshold

FN = number of true positive matches with scores < threshold

TN = number of false positive matches with scores < threshold

Using these values, we generated receiver operating characteristic (ROC) curves and calculated the area under the curve (auROC) to summarize overall classification accuracy. However, ROC curves and their associated FPRs can be misleading in imbalanced datasets, where a large number of true negatives deflates the FPR even when the absolute number of false positives is high. In contrast, the FDR measures the proportion of predicted positives that are actually false at a given threshold, providing a more informative measure of performance in an unbalanced dataset. For this reason, we focused on **FDR-TPR curves** across SimPheny Score thresholds to benchmark the behavior and accuracy of the SimPheny algorithm.

## Supplementary Material

Supplement 1

## Figures and Tables

**Table 1:** Summary statistics for SimPheny Matches across varying SimPheny Score thresholds in benchmarking and discovery cohorts.

SimPheny Score	Undiagnosed Matches	TP Matches	FP Matches	TPR	FDR	SimPheny Matches	SimPheny Candidates	Confidence
≥ 6.0	0	3	0	1.1%	0.0%	1	1	High
≥ 5.5	1	11	0	4.1%	0.0%	1	1	High
≥ 5.0	5	29	4	10.7%	12.1%	1	1	High
**≥ 4.5**	**18**	**51**	**11**	**18.8%**	**17.7%**	**1**	**1**	**High**
≥ 4.0	80	79	30	29.2%	27.5%	1	1	Medium
≥ 3.5	185	110	85	40.6%	43.6%	1	1	Medium
≥ 3.0	425	145	191	53.5%	56.8%	2	1	Medium
≥ 2.5	899	185	400	68.3%	68.4%	2	2	Medium
≥ 2.0	2025	221	825	81.5%	78.9%	3	2	Low
≥ 1.5	4407	251	1480	92.6%	85.5%	4	3	Low
≥ 1.0	7351	265	2266	97.8%	89.5%	6	4	Low
≥ 0.5	9948	270	2882	99.6%	91.4%	8	5	Low
≥ 0.0	11481	271	3171	100%	92.1%	9	5	Low

SimPheny was run on 1,851 UDN probands - 404 diagnosed individuals in the benchmarking cohort and 1,447 undiagnosed individuals in the discovery cohort - querying against a reference dataset of 767 diagnosed UDN participants (Supplementary Fig. 1). Each row summarizes results at a given SimPheny Score threshold in the leftmost column. True positive (TP) and false positive (FP) matches were determined in the benchmarking cohort of 404 diagnosed probands with known diagnostic genes. “Undiagnosed Matches” refers to matches returned from the 1,447 undiagnosed probands. TPR (true positive rate) and FDR (false discovery rate) were calculated from the benchmarking data (Fig. 3). “SimPheny Matches” and “SimPheny Candidates” indicate the average number of matches or candidate genes per proband (of 1,851) among those with at least one match at each score threshold. Confidence tiers were derived from benchmarking performance.

**Table 2:** High-confidence candidate gene matches (SimPheny Score ≥ 4.5) in undiagnosed UDN probands.

Proband	PhenoSim_Jaccard_	Candidate Gene	SimPheny Score	Status
UDN1	0.352	RPL13	6.091	Diagnostic
UDN1	0.314	RPL13	5.246	Diagnostic
UDN2	0.464	AFG3L2	5.206	Contemporaneously Diagnosed
UDN3	0.316	KIF5B	5.050	Contemporaneously Diagnosed
UDN4	0.417	MED12	5.003	Unresolved
UDN5	0.660	SET	4.960	Unresolved
UDN6	0.326	HCFC1	4.904	Unresolved
UDN7	0.380	WDR73	4.896	Unresolved
UDN8	0.384	WARS2	4.786	Contemporaneously Diagnosed
UDN9	0.405	TRAF3	4.780	Rejected
UDN10	0.498	MTOR	4.750	Unresolved
UDN11	0.506	ANK3	4.744	Unresolved
UDN12	0.344	MED12	4.727	Unresolved
UDN13	0.513	ERCC4	4.711	Unresolved
UDN14	0.316	GLUL	4.631	Contemporaneously Diagnosed
UDN15	0.340	SPTBN1	4.562	Tentative Diagnosis
UDN16	0.301	RFC1	4.548	Unresolved
UDN17	0.262	UBA1	4.538	Contemporaneously Diagnosed

SimPheny was applied to 1,447 undiagnosed UDN probands in the discovery cohort, querying against a reference dataset of 767 diagnosed UDN participants (Supplementary Fig. 1). Each row represents a high-confidence SimPheny Match, including the phenotypic similarity score (PhenoSim_Jaccard_), SimPheny Score, and classification status based on manual review. “Diagnostic” refers to candidates newly identified by SimPheny that are now considered diagnostic by the UDN clinical team. “Contemporaneously Diagnosed” matches correspond to diagnoses independently reached by UDN sites but not recorded in the UDN internal database at the time of cohort curation. “Tentative Diagnosis” denotes a match in which the candidate gene was recorded in the UDN internal database but excluded from our diagnosed cohort due to diagnostic uncertainty (Methods). “Rejected” candidates were ruled out by UDN clinical review. “Unsolved” candidates refer to cases where SimPheny candidates lack sufficient evidence to be either rejected or confirmed, although we note that some have benign/likely benign variant classifications in ClinVar. See Supplementary Table 3 for extended match details.

## Data Availability

All deidentified exomic and genomic sequencing data, including SNV and indel variant calls, as well as corresponding phenotype data in the form of pedigree files and HPO terms, are regularly deposited in dbGaP (accession phs001232.v7.p3). Rare SNV and indel variants, as well as HPO terms, for UDN participants with genomic sequencing are also queryable in a public-facing browser: https://dbmi-bgm.github.io/udn-browser/. Variant-level data, clinical significance, and supporting evidence, as well as demographic information and phenotype information for all diagnostic variants in the UDN, are periodically submitted to ClinVar. Other relevant, patient-specific clinical information may be shared on a case-by-case basis at the discretion of the clinical team managing the case. Requests for such data can be directed to udncc@hms.harvard.edu with a response expected within six weeks.

## Code Availability

SimPheny.iobio is freely available as an open-source web application at https://github.com/iobio/SimPheny.iobio. For cohort-scale analyses, SimPheny can also be run locally via https://github.com/icooperstein/SimPheny. All code used to perform the analyses, generate figures, and support the findings of this manuscript is available in the same repository.
